# Identification of Antidiabetic Metabolites from* Paederia foetida* L. Twigs by Gas Chromatography-Mass Spectrometry-Based Metabolomics and Molecular Docking Study

**DOI:** 10.1155/2019/7603125

**Published:** 2019-05-29

**Authors:** Dai Chuan Tan, Nur Kartinee Kassim, Intan Safinar Ismail, Muhajir Hamid, Muhammad Safwan Ahamad Bustamam

**Affiliations:** ^1^Department of Chemistry, Faculty of Science, Universiti Putra Malaysia, 43400 UPM Serdang, Selangor, Malaysia; ^2^Laboratory of Natural Product, Institute of Bioscience, Universiti Putra Malaysia, 43400 UPM, Serdang, Selangor, Malaysia; ^3^Department of Microbiology, Faculty of Biotechnology and Biomolecular Sciences, Universiti Putra Malaysia, 43400 UPM Serdang, Selangor, Malaysia

## Abstract

*Paederia foetida* L. (Rubiaceae) is a climber which is widely distributed in Asian countries including Malaysia. The plant is traditionally used to treat various diseases including diabetes. This study is to evaluate the enzymatic inhibition activity of* Paederia foetida* twigs extracts and to identify the metabolites responsible for the bioactivity by gas chromatography-mass spectrometry (GC-MS) metabolomics profiling. Three different twig extracts, namely, hexane (PFH), chloroform (PFC), and methanol (PFM), were submerged for their *α*-amylase and *α*-glucosidase inhibition potential in 5 replicates for each. Results obtained from the loading column scatter plot of orthogonal partial least square (OPLS) model revealed the presence of 12 bioactive compounds, namely, dl-*α*-tocopherol, n-hexadecanoic acid, 2-hexyl-1-decanol, stigmastanol, 2-nonadecanone, cholest-8(14)-en-3-ol, 4,4-dimethyl-, (3*β*,5*α*)-, stigmast-4-en-3-one, stigmasterol, 1-ethyl-1-tetradecyloxy-1-silacyclohexane, ɣ-sitosterol, stigmast-7-en-3-ol, (3*β*,5*α*,24S)-, and *α*-monostearin.* In silico* molecular docking was carried out using the crystal structure *α*-amylase (PDB ID: 4W93) and *α*-glucosidase (PDB ID: 3WY1). *α*-Amylase-n-hexadecanoic acid exhibited the lowest binding energy of -2.28 kcal/mol with two hydrogen bonds residue, namely, LYS178 and TYR174, along with hydrophobic interactions involving PRO140, TRP134, SER132, ASP135, and LYS172. The binding interactions of *α*-glucosidase-n-hexadecanoic acid complex ligand also showed the lowest binding energy among 5 major compounds with the energy value of -4.04 kcal/mol. The complex consists of one hydrogen bond interacting residue, ARG437, and hydrophobic interactions with ALA444, ASP141, GLN438, GLU432, GLY374, LEU373, LEU433, LYS352, PRO347, THR445, HIS348, and PRO351. The study provides informative data on the potential antidiabetic inhibitors identified in* Paederia foetida* twigs, indicating the plant has the therapeutic effect properties to manage diabetes.

## 1. Introduction 

Diabetes mellitus is a chronic metabolic disorder of the pancreas often referred to simply as diabetes and is characterized by highly elevated blood glucose levels with disturbances in carbohydrate, fat, and protein metabolism [1]. It occurs either due to defective insulin secretion by the pancreas (i.e., the pancreas does not produce enough insulin) or due to the ineffective response by the cells to the insulin that is produced. World Health Organization (WHO) stated that an estimated 422 million adults were living with diabetes in 2014 compared to 108 million in 1980 [2]. The number of people with diabetes has nearly doubled since 1980, increasing from 4.7% to 8.5% in the adult population due to being overweight or obese. Prevalence of diabetes is rising faster in low- and middle-income countries than in high-income countries. Diabetes triggered 1.5 million deaths globally in 2012 [2].

*Paederia foetida* L. or skunk vine, locally known as “Daun Sekentut,” has antidiabetic properties. It is a climber widely distributed in Asian countries including Malaysia, Thailand, China, Vietnam, etc. [3]. It can be eaten raw which is commonly practised in Malaysia. The plant has been traditionally used to treat sores, rheumatic joint, night blindness, digestive problems, toothache, etc. [4]. In addition,* P. foetida* is good also for women after childbirth [5]. The previous study of the plant showed it has some bioactivities, such as anti-inflammatory [4], antinociceptive [6], antidiarrheal [7], antioxidant [5, 8], antihepatotoxic [9], antidiabetic [3, 10], antitussive [11] and gastroprotective [8] activities.

Metabolomics is a tool to identify the bioactive markers of the medicinal plants. It is also to quantify all the metabolites present in a biological system under a particular condition [12]. It is also a holistic approach that includes the detection of all metabolites in each sample and can correlate to the bioactivity using multivariate data analysis (MVDA) [13]. The common methods in metabolomics such as gas chromatography-mass spectrometry (GC-MS), nuclear magnetic resonance spectrometry (NMR), and liquid chromatography-mass spectrometry (LC-MS) [14]. For the MS-based nontargeted metabolomics study, the crude extracts from different solvents were analyzed by a collection of chemical structures including retention time, area percentage, mass-to-charge ratios (m/z), and the similarity index of each metabolite. Nontargeted metabolomics study employs data processing algorithms for aligning bulky datasets and providing information on all detectable m/z [15]. The resulting data matrix is significant for a comparison of chemical profiles among different extracts using multivariate data analysis tools.

The study of* Paederia foetida* twigs as an antidiabetic agent is very limited especially on the bioactive compounds responsible for the biological property of the plant. Therefore, the objectives of this study are to identify the bioactive compounds from the* Paederia foetida* twigs extract as an antidiabetic agent using metabolomics approach. MVDA is a suitable statistical tool for managing large data sets obtained using spectroscopic tools and is employed in classifying samples based on their phytoconstituents [16].

## 2. Material and Methods

### 2.1. Instrument and Chemical Reagents

The gas chromatography-mass spectrometry (GC-MS) of the extracts was recorded by using a Shimadzu model QP5050A with BPX5 for nonpolar (5% phenylmethylsilane) capillary column (30 m × 250 *μ*m × 0.25 *μ*m). The antidiabetic assay was performed using *μ*-QUANT model microplate reader. The *α*-amylase and *α*-glucosidase enzymes were purchased from Megazyme. The* p*-nitrophenyl *α*-d-glucopyranoside, soluble starch, potassium sodium tartrate, 3,5-di-nitro salicylic acid (DNS), sodium hydroxide, dimethyl sulfoxide (DMSO), and other chemicals and solvents of analytical grade were purchased from Sigma.

### 2.2. Plant Materials

*P. foetida* was collected from Ledang, Johor in Malaysia on 7th June 2017. The plant sample was submitted to Institute of Bioscience (IBS), Universiti Putra Malaysia (UPM), Serdang, for plant identification which gave the specimen voucher number of SK3177/17. The twigs of the plant were dried at room temperature and ground into powder.

### 2.3. Extraction Method

The powdered twigs were extracted using hexane, chloroform, and methanol solvents individually. A total of 15 plant extracts was obtained from 5 biological replicates of each extraction solvent. The extraction was performed by weighing 50 g of ground samples, mixing them with 200 mL of hexane in a 500 mL conical flask and subjecting to soaking for 72 hours. The solvent suspension was filtrated and concentrated using a rotary evaporator to yield the crude extract. The crude extracts were stored in an amber bottle at 4°C until further analysis. Thus, the chloroform and methanol were applying the same extraction method as above. All 15 replicates of extracts were subjected to enzyme inhibition assays (*α*-amylase and *α*-glucosidase), and the metabolites were analyzed by using GC-MS.

### 2.4. Enzymatic Assays

#### 2.4.1. *α*-Amylase Inhibition

The *α*-amylase inhibition was determined using the iodine-starch test [17]. 60 *μ*L of 0.1 M sodium phosphate buffer, 20 *μ*L of 1 U/mL *α*-amylase, and 50 *μ*L extract at concentration within 0.078-5 mg/mL were mixed in the well and incubated for 15 min at 37°C. After that, a 50 *μ*L of 0.5% soluble starch was then added to the reaction mixture and incubated for 15 min at 37°C. After incubation, 20 *μ*L of 1 M hydrochloric acid was added to stop the enzyme activity. The mixture was incubated in a hot water bath for 5 min. Lastly, 50 *μ*L of iodine reagent was added to the reaction mixture and the absorbances were taken at 620 nm wavelength. Acarbose and distilled water were used as positive and negative controls. The activity was calculated using (1) and (2) as follows:

(1) Relative  α-amylase  enzyme  activity  %=enzyme activity of sampleenzyme activity of negative control×100%

(2) α-amylase enzyme inhibition %=100%−Relative  α-amylase enzyme activity %

The IC_50_ values were determined by the calibration curve of *α*-amylase inhibition against the concentration of extracts.

#### 2.4.2. *α*-Glucosidase Inhibition

*α*-Glucosidase inhibition assay was performed according to Collins* et al.* [18], Deautschländer* et al.* [19], and Sajak* et al.* [20] with slight modification. The reaction mixtures consisting 10 *μ*L of the test sample (0.078-5 mg/mL), 130 *μ*L of 30 mM of phosphate buffer solution (pH 6.5), and 10 *μ*L of *α*-glucosidase working solution were incubated at 37°C for 30 min. After incubation, 50 *μ*L of p-nitrophenyl-*α*-d-glucopyranoside (50 mM phosphate buffer, pH 6.5) was added into the well, and the reaction mixtures were incubated for another 30 min at 37°C. Lastly, 50 *μ*L of glycine (2 M, pH 10) was added to the mixtures for termination of the reaction. The 405 nm of wavelength was used for reading of results. The calculation of *α*-glucosidase inhibition activity of the test samples using (3) is as follows:

(3) $$\begin{array}{c} \%\;\;Inhibition=\left(\;∆Ac-\frac{∆Ae}{∆Ac}\;\right)\times 100\% \end{array}$$

whereas Δ A_c_ is the absorbance difference between negative control and blank negative control while Δ A_e_ is the absorbance difference between the test sample and blank test sample. Acarbose and DMSO were used as positive and negative control.

### 2.5. GC-MS Analysis

The profiling of nontargeted metabolites was performed using GC-MS QP2010 Plus SHIMADZU [13]. At a temperature of 230°C and using the split injection mode (ratio of 3:1), 1 *μ*L of the sample was loaded into the sample inlet unit (GC) coupled with a mass spectrometer detector. A ZB-5MS column with an inner diameter (ID) of 30 m × 0.25 mm and a film thickness of 0.25 *μ*m was used for this analysis. The initial column oven temperature was set to 40°C for 3 min and then increased to a target temperature of 325°C in 10 min at a rate of 10°C/min. The mass spectra were acquired over a mass scan range of 35 to 700 m/z. The spectra and retention times generated were compared with those of known chemical compounds libraried in the NIST08 database library. Perfluorotributylamine, an internal standard, was used in this analysis. The solution of the internal standard was injected into and analyzed by the GC-MS.

### 2.6. Data Processing and Statistical Analysis

All results are expressed as mean ± standard deviation and differences between means were statistically analyzed using the t-test for comparison between two treatments. p < 0.05 was considered significant. Prior to multivariate data analysis, XCMS is a package developed in R programming and made available by Bioconductor Project for the treatment of MS data. In this analysis, the version 3.3.2 of XCMS package in R was used for data processing. The raw GC-MS data was converted into computable document format (.cdf) prior to the XCMS analysis. The GC-MS data were binned into components from 39.049-415.3506* m/z* in 218.5-1287.75-second run time. The results were organized into tabular dataset format with retention times (rows) against peak intensities (columns). The characteristic mass to charge (m/z) ratios and retention times of compounds resulting from the GC-MS analysis were compared with those of standards stored in the NIST08 spectral database. Based on the mass spectral pattern comparison, the molecular weight, names, and structures of all the compounds were determined. Once after XCMS processing, the data was loaded to SIMCA 14.1 software (version 14.1, Umetrics, Umeå, Västerbotten, Sweden) using Principal Component Analysis (PCA), Partial Least Square (PLS), and Orthogonal Partial Least Square (OPLS) via the method of UV scaling. The fitness and validation of the model were done through the permutation and ANOVA tests. Finally, the score scatters and loading plots were produced.

### 2.7. *In Silico* Molecular Docking

The *α*-amylase and *α*-glucosidase were analyzed and downloaded from Protein databank with PDB ID 4W93 and 3WY1, respectively. The AutoDock 4.2 was used for* in silico* molecular docking of protein-ligand interactions [21]. The removal of unwanted chains, crystal water, and nonpolar hydrogen atoms and the addition of hydrogen atoms in the receptor were carried out. Then, the MGAM protein structure was removed from these receptors, thus subjected to docking study. The molecular structure of ligand was recouped from ChemDraw 16.0. The Gasteiger charges and hydrogen atoms were added to the ligand via AutoDock 4.2. Docking Grid Box was used for calculating the grid maps and centred on the ligand or compound coordinate with 28 Å × 28 Å × 28 Å dimension and a grid spacing of 1 Å [22]. The docking results were analyzed using Discovery Studio Visualizer software.

## 3. Results and Discussion

### 3.1. Enzymatic Activity

The *α*-amylase and *α*-glucosidase inhibition activity of* P. foetida* twigs extracts obtained from different solvent extracts are displayed in Table 1 as half maximal concentration values (IC_50_, *μ*g/mL). A lower IC_50_ value is favoured to higher enzymatic activity. The highest *α*-amylase and *α*-glucosidase inhibition activity were observed for chloroform extract with the IC_50_ value of 600.287 ± 0.06 and 1349.01 ± 0.01 *μ*g/mL, respectively. The positive control, acarbose, was used in the study.

### 3.2. GC-MS Metabolomic and Multivariate Data Analysis

**Figure 1:**
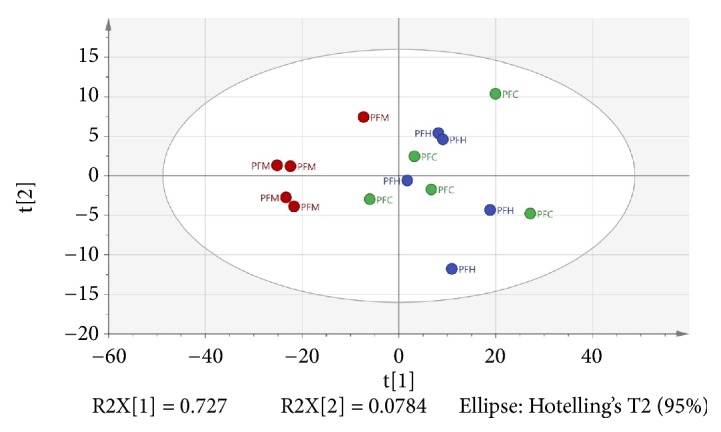
PCA score plot of plant extracts based on GC-MS spectra. PFH, PFC, and PFM are hexane, chloroform, and methanol extracts, respectively.

A total of 397 presumptive compounds were detected using GC-MS metabolomics. 12 of the 397 compounds were interpreted as plant metabolites using retention times, area percentage, and similarity index (Table 2). The bioactive compounds were identified through GC-MS metabolomics utilizing MVDA whereby PCA, PLS, and OPLS model were developed. Automated fitting results in two principal components, principal components 1 and 2. The certain retention time was extracted based on the ratio of mass to charge (m/z) and the IC_50_ (*μ*g/mL) values of each of the samples were considered as x and y variables, respectively. PCA was applied to the data to observe the possible presence of trends and groupings which were previously not obvious by looking at the data. PCA allowed us to detect outliers too [23]. The results of the PCA showed outliers were not present among the samples as all observations were situated within the normal range of the Hoteling T^2^ ellipse (Figure 1). In this study, GC-MS dataset was subjected to PCA to understand the clustering characteristic of the hexane (PFH), chloroform (PFC), and methanol (PFM) extracts and to determine the compounds responsible for their discrimination. There is no distinct discrimination of PFH with PFC. However, PFM could be clearly distinguished from other groups of extracts. The resulted model showed good fitness and high predictability with R^2^X = 0.952 and Q^2^ = 0.802, respectively.

To understand the relationship between studied bioactivities and the plant extracts, PLS as a supervised multivariate data was applied. PLS provides a correlation between metabolites and bioactivities. Thus metabolites that were playing roles as phytochemical markers may be suggested. The model diagnostic of the PLS model developed can be evaluated using several parameters such as goodness of fit, a test of permutation, and the capability of the model to predict the value using actual to predicted plot [18]. The cumulative values of R2Y explain the goodness of fit is indicating the percentage of variation of the response explained by the model, and the cumulative value of Q^2^Y is representing the percentage of the variation of the response predicted by the model according to cross-validation [13]. The fitness of model and predictive ability is considered good if the cumulative values of both R^2^Y and Q^2^Y are greater than 0.5 [24].

**Figure 2:**
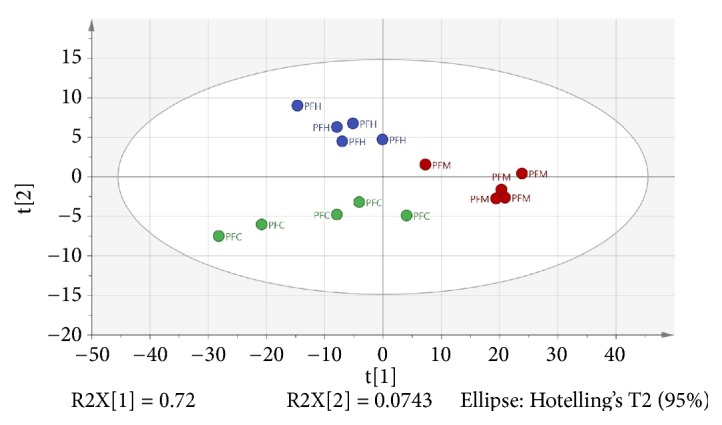
PLS score plot of plant extracts based on GC-MS spectra. PFH, PFC, and PFM are hexane, chloroform, and methanol extracts, respectively.

**Figure 3:**
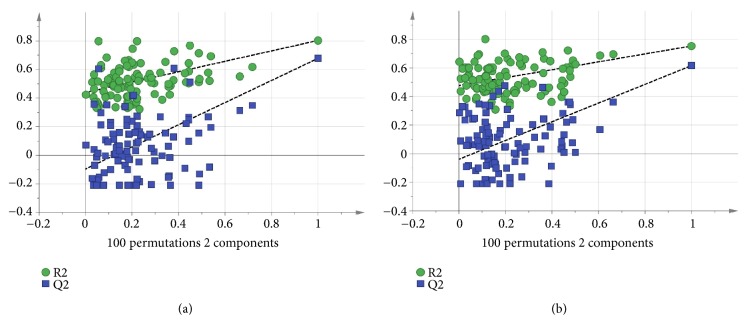
(a) The permutation test for the two components of the PLS model with R^2^Y = 0.439 and Q^2^Y = -0.0958 for *α*-amylase. (b) The permutation test for the two components of the PLS model with R^2^Y = 0.477 and Q^2^Y = -0.038 for *α*-glucosidase.

Based on Figure 2, the distinct discrimination among the three extracts is clearly showed which correlated to the bioactivities. The resulted model also showed good fitness and prediction value with R^2^Y = 0.778 and Q^2^ = 0.647, respectively, demonstrating that this PLS model is good. The PLS model was further validated using internal cross-validation by means of R^2^Y and Q^2^ cumulative and permutation tests with 100 permutations. Permutation plots of the PLS model are shown in Figure 3. The *α*-amylase-intercepts and *α*-glucosidase-intercepts of R^2^ were 0.439 and 0.477, respectively. The PLS model had a significant difference (p < 0.05) in correlation with *α*-amylase *α*-glucosidase inhibition with the p-values of 0.01 and 0.03. Therefore, the PLS model met the criteria for a good performance model. Figure 2 showed the score scatter plot of the PLS model with good separation of the samples based on the chemical profile and bioactivity of the plant extracts.

**Figure 4:**
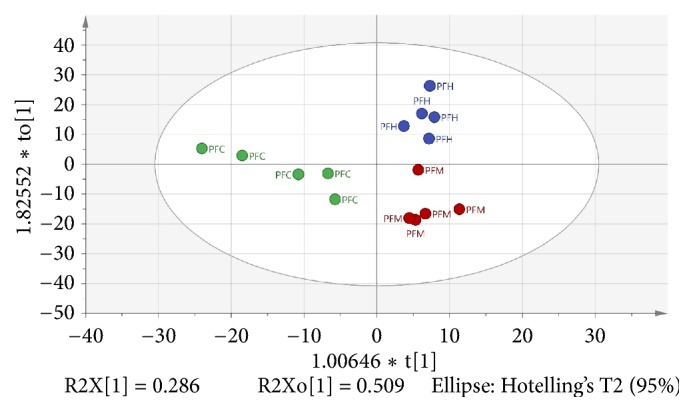
OPLS score plot of plant extracts based on GC-MS spectra. PFH, PFC, and PFM are hexane, chloroform, and methanol extracts, respectively.

**Figure 5:**
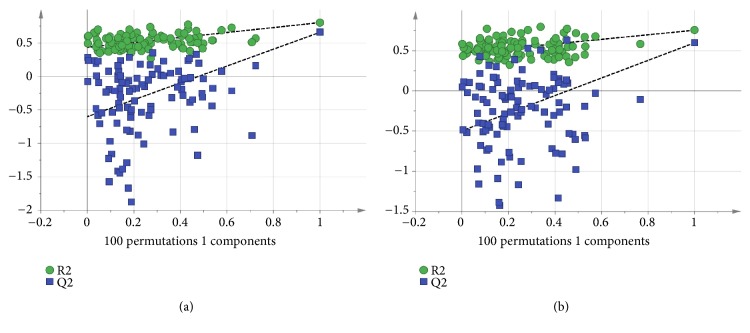
(a) The permutation test for the one component of the OPLS model with R^2^Y = 0.433 and Q^2^Y = -0.601 for *α*-amylase. (b) The permutation test for the one component of the OPLS model with R^2^Y = 0.47 and Q^2^Y = -0.496 for *α*-glucosidase.

Besides, OPLS is another supervised multivariate data technique used to derive a correlation between the extracts and bioactivities. Based on Figure 4, there was distinct discrimination of the three extracts correlated to bioactivities among each other. The model also showed good fitness and prediction value with R^2^Y = 0.778 and Q^2^ = 0.628, respectively, demonstrating that this OPLS is a good model. The OPLS model was further validated using internal cross-validation by means of R^2^ and Q^2^ cumulative and permutation tests with 100 permutations. Permutation plots of the OPLS model are shown in Figure 5. The *α*-amylase-intercepts and *α*-glucosidase-intercepts of R^2^ were 0.433 and 0.47, respectively. The OPLS model had a significant difference (p < 0.05) in correlation with *α*-amylase *α*-glucosidase inhibition with the p-values of 0.02 and 0.04. Therefore, the OPLS model meets the criteria of a good performance model. The active PFC extract was observed at the negative OPLS component 1, while the less active plant extracts, PFH and PFM extracts, were distributed at the positive OPLS component 1.

**Figure 6:**
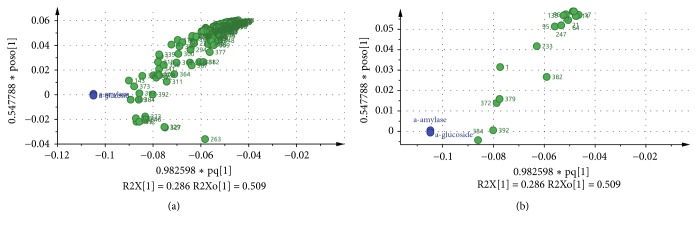
OPLS loading scatters plot of active extract in the range -0.1 to -0.02. (a) All the peak numbers on GC-MS chromatogram. (b) Selected peak numbers on GC-MS chromatogram.

As evident in Figure 4, sample groupings were observed from the OPLS score plot; PFC was distinguished from PFH and PFM. From the score plot, PFC contributed significantly to the variations along with principal component 1 (PC1), as it is the only sample situated on the left quadrant. The OPLS loading scatters plot (Figure 6) displays the relationship between the x variables which are the GC-MS data and the variables which are the activities. It is obvious from the loading plot that the variables at the far left in the top quadrant by PC1 are correlated to the observed bioactivities.

**Figure 7:**
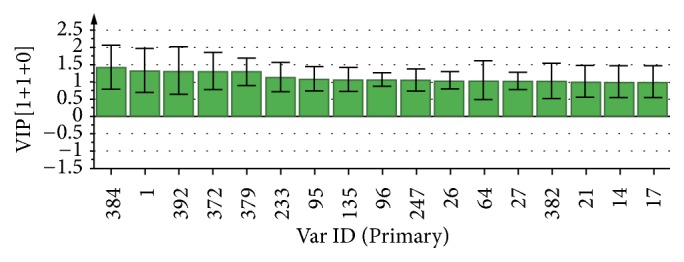
VIP plot of active extract of* P. foetida* twigs. Metabolites identification (Var ID): (C29H50O: 1, 96, 379, 383); (C16H32O2: 14, 17, 64); (C16H34O: 21); (C29H52O: 26, 27, 135); (C19H38O: 95); (C29H48O: 233, 247); (C21H44OSi: 372,392) and (C21H42O4: 384).

To identify that the putative compounds may contribute to the bioactivities, the Variable Importance of Projection (VIP) plot from the OPLS model was surveyed, and potential bioactive compounds were selected from the results of the VIP analysis (Figure 7). Important x variables are those with VIP values that are larger than the average VIP of 1.0 [25]. Therefore, the VIP plot was sorted to eliminate the variables with values less than 1.0 and a list of important variables were afterwards generated. These were further sorted, and the identities of these important variables were determined by tracing the retention times to those in the total ion chromatogram (TIC). Each of the peaks indicates the identified metabolite from the* P. foetida* twigs active extracts and the details of the compound have been tabulated in Table 2. The 17 peaks are assumed to induce *α*-amylase and *α*-glucosidase activity. These compounds were found abundant in the active chloroform extract. Further comparison with NIST08 spectral database confirmed that the 17 peaks correspond to dl-*α*-tocopherol, n-hexadecanoic acid, 2-hexyl-1-decanol, stigmastanol, 2-nonadecanone, cholest-8(14)-en-3-ol, 4,4-dimethyl-, (3*β*,5*α*)-, stigmast-4-en-3-one, stigmasterol, 1-ethyl-1-tetradecyloxy-1-silacyclohexane, ɣ-sitosterol, stigmast-7-en-3-ol, (3*β*,5*α*,24S)-, and *α*-monostearin (Table 2). In accordance with the results shown in Table 2, there are five bioactive compounds showing high abundance in the active extract, n-hexadecanoic acid, cholest-8(14)-en-3-ol, 4,4-dimethyl-, (3*β*,5*α*)-, ɣ-sitosterol, stigmast-7-en-3-ol, (3*β*,5*α*,24S)-, and stigmasterol with the values of 29.48, 20.20, 20.20, 20.20, and 10.51%. Therefore, these five compounds contributed more to the antidiabetic properties of* Paederia foetida* compared to other compounds.

### 3.3. *In-Silico* Molecular Docking

Molecular docking was carried out in order to understand the interaction between protein and ligand [13]. Protein is commonly comprised of amino acids linked in a sequence to form the complex, which can be hydrophobic, polar, or electrically charged form. This has been the key to the actual properties and behaviours of a protein [26]. The main five bioactive compounds identified to be actively responsible for the *α*-amylase and *α*-glucosidase inhibition were docked to the *α*-amylase and *α*-glucosidase crystal structure (PDB ID: 4W93 and 3WY1). The conformations showing the lowest binding energy for the compounds with the interacting residues are summarized in Tables 3 and 4.

**Figure 8:**
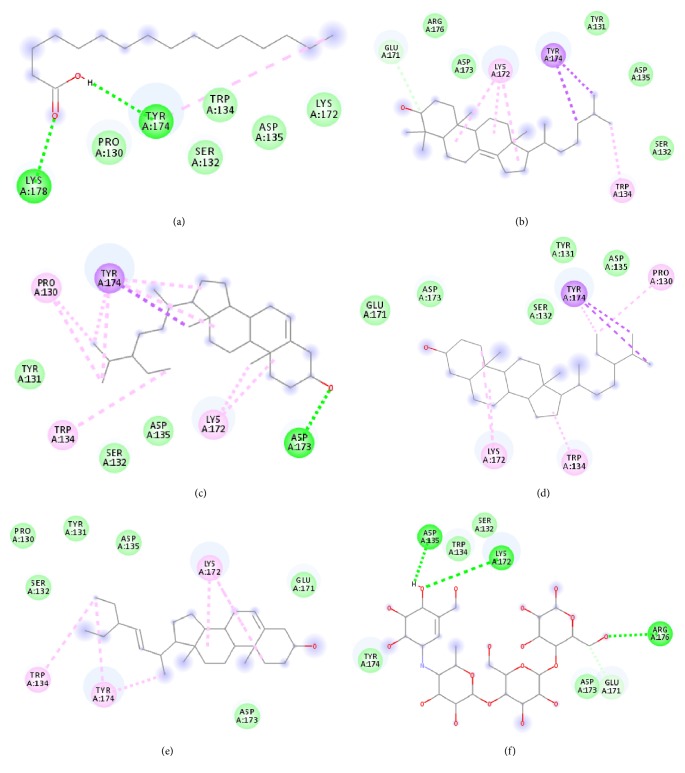
The 2D diagram showing the interaction between the protein residues of *α*-amylase and the inhibitors. (a) n-hexadecanoic acid, (b) cholest-8(14)-en-3-ol, 4,4-dimethyl-, (3*β*,5*α*)-, (c) ɣ-sitosterol, (d) stigmast-7-en-3-ol, (3*β*,5*α*,24S)-, (e) stigmasterol, (f) acarbose.

Molecular docking was run using AutoDock 4.2 with Lamarckian Genetic Algorithm (Lamarckian GA), and some of the evaluations were selected in the long category with RMSD 2.0 Å. Based on the AutoDock 4.2 simulation result of *α*-amylase shown in Table 3, *α*-amylase-acarbose inhibitor complex showed +1.31 kcal/mol binding energy containing three hydrogen bonds with the interacting residues, ASP135, LYS172, ARG176, and hydrophobic interactions with TRP134, SER132, TYR174, ASP173, and GLU171. Meanwhile, n-hexadecanoic acid exhibited -2.28 kcal/mol with the *α*-amylase in the complex ligand. A total of two hydrogen bonds were observed in the complex ligand, namely, LYS178 and TYR174, along with hydrophobic interactions involving PRO140, TRP134, SER132, ASP135, and LYS172. ɣ-Sitosterol showed -5.28 kcal/mol in the complex with only one hydrogen bond, ASP173, along with eight hydrophobic interactions, ASP135, GLU171, SER132, TYR131, LYS172, PRO130, TRP134, and TYR174. Despite no interaction with residue by a hydrogen bond with its hydroxyl group, stigmasterol has comparably shown better binding energy of -5.35 kcal/mol than the positive control, acarbose. The hydrophobic contacts appeared to be leading in *α*-amylase-stigmasterol complex ligand due to its cyclic skeleton and alkyl groups that preferable bind to ASP135, ASP173, GLU171, PRO130, SER132, TYR131, LYS172, TRP134, and TYR174. Stigmasterol was observed to be destined near to the active site which is similar binding interactions to the acarbose (Figure 8). Its binding is mainly assisted through hydrophobic contacts that contribute to its binding energy thus reflecting its inhibition activity. So, it might slow down the enzyme activity. The stigmasterol's predicted binding part could be a promising region for inhibiting this protein activity overall through a competitive mode [13]. Besides, cholest-8(14)-en-3-ol, 4,4-dimethyl-, (3*β*,5*α*)-, and stigmast-7-en-3-ol, (3*β*,5*α*,24S)- also showed no interaction with hydrogen bond residue with the binding energy of -5.10 and -4.98 kcal/mol. In addition, due to a lack of hydroxyl groups in the tested compounds, the hydrophobic and *π*-interactions apparently play an important role for these compounds' enzyme inhibition activity.

**Figure 9:**
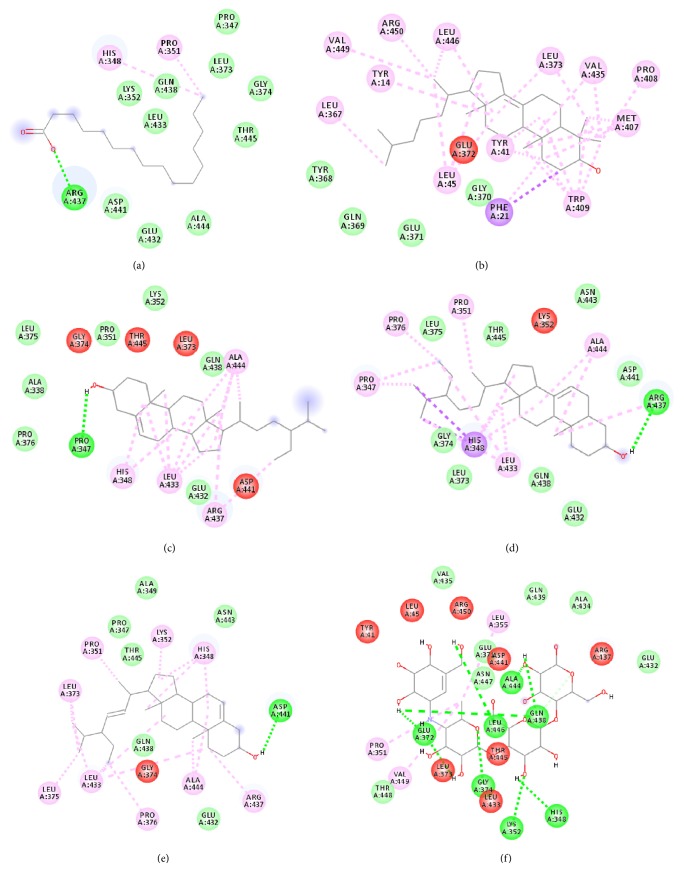
The 2D diagram showing the interaction between the protein residues of *α*-glucosidase and the inhibitors. (a) n-hexadecanoic acid, (b) cholest-8(14)-en-3-ol, 4,4-dimethyl-, (3*β*,5*α*)-, (c) ɣ-sitosterol, (d) stigmast-7-en-3-ol, (3*β*,5*α*,24S)-, (e) stigmasterol, (f) acarbose.

The binding interactions of *α*-glucosidase-n-hexadecanoic acid complex ligand showed the lowest binding energy among the other 5 compounds with -4.04 kcal/mol (Table 4). Based on Figure 9, the complex consists of one hydrogen bond interacting residue, ARG437, and hydrophobic interactions with ALA444, ASP141, GLN438, GLU432, GLY374, LEU373, LEU433, LYS352, PRO347, THR445, HIS348, and PRO351. Stigmast-7-en-3-ol, (3*β*,5*α*,24S)- in complex ligand contained the same hydrogen bond interacting residue to n-hexadecanoic acid complex ligand with binding energy of +32.55 kcal/mol and along with hydrophobic interactions of ASN443, ASP441, GLN438, GLU432, GLY374, LEU373, LEU375, THR445, ALA444, LEU433, PRO347, PRO351, PRO376, and HIS348. At the same time, the binding energy of acarbose in the complex ligand with *α*-glucosidase was +2547.97 kcal/mol. A total of seven hydrogen bonds were observed in the complex ligand involving ALA444, GLN438, GLU372, GLY374, HIS348, LEU446, and LYS352 along with hydrophobic interactions involving ALA434, ASN447, GLN439, GLU371, GLU432, THR448, VAL435, LEU355, PRO351, and VAL449. Besides, *α*-glucosidase-ɣ-sitosterol complex ligand showed a binding affinity value of +10.38 kcal/mol. The PRO347 residue was observed to interact with ɣ-sitosterol via a hydrogen bond, while ALA338, GLN438, GLU432, LEU375, LYS352, PRO351, PRO376, ALA444, ARG437, HIS348, and LEU433 were interacting hydrophobically. Then hexadecanoic acid has shown comparable binding energy compared to acarbose and other compounds. It is due to the absence of unfavourable interactions (red colour) in the complex ligand. The unfavourable interaction was strongly influencing the molecular binding energy, thus causing the ligands and receptors to be not so stable. The unfavourable interactions between atom pairs were determined as close contacts relative to the* van der Waals* distance between atoms. A steric bump happens when the atom-atom distance is less than or equal to a threshold expressed as a fraction of the sum of the atoms' van der Waals radii. So, acarbose contains many unfavourable steric interactions which make the complex ligand to be more unstable and require larger binding energy.

## 4. Conclusion

The metabolomics statistical analysis data showed that the experimental result is a good model in PLS and OPLS with the acceptable R^2^ and Q^2^ values. Stigmasterol and n-hexadecanoic acid are suggested to be the enzyme inhibitors metabolites in the twigs of* Paederia foetida* as *α*-amylase-stigmasterol, and *α*-glucosidase-n-hexadecanoic acid complexes showed low binding energy of -5.35 and -4.04 kcal/mol. The molecular docking study helps to understand and visualize the virtual scenario of the binding interaction of the plant metabolites with the selected enzymes at the molecular level. Further, the* in-vitro* enzymatic assays of the 5 major compounds in the plant extract should be carried out to validate the* in-silico* results.

## Figures and Tables

**Table 1:** The half maximal inhibitory concentration (IC_50_) of antidiabetic inhibition activity of *P. foetida* twigs extracts.

Samples	*α*-amylase inhibition activity IC_50_ (*μ*g/mL)	*α*-glucosidase inhibition activity IC_50_ (*μ*g/mL)
Hexane	4553.403 ± 0.04	9143.469 ± 0.03

Chloroform	600.287 ± 0.06	1349.01 ± 0.01

Methanol	> 10 000	> 5 000

Acarbose	10.57 ± 0.01	0.056 ± 0.01

Data expressed as mean ± standard deviation (n = 5). Means that do not share a letter are significantly different with p value < 0.05.

**Table 2:** The bioactive compounds identified in *Paederia foetida* chloroform extract.

Peak No.	RT (min)	Area Percentage (%)	Chemical formula & Molecular weight (g/mol)	Similarity index (%)	Compound Name and structure
1	18.258	0.96	C_29_H_50_O (430)	86	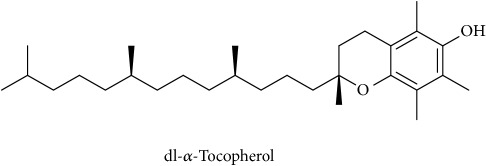

14, 17, 64	13.433	29.48	C_16_H_32_O_2_ (256)	97	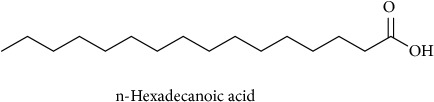

21	13.433	0.85	C_16_H_34_O (242)	85	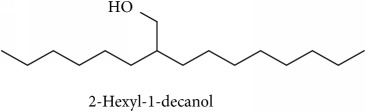

26, 27, 135	19.442	0.54	C_29_H_52_O (416)	81	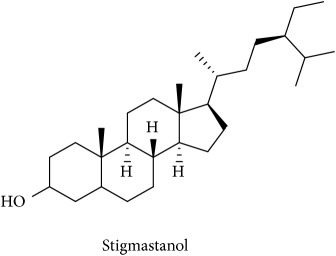

95	15.858	1.35	C_19_H_38_O (282)	93	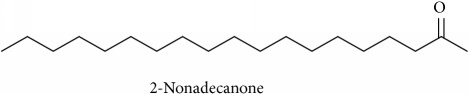

96	19.408	20.20	C_29_H_50_O (414)	77	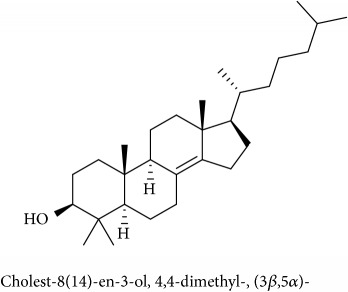

233	20.233	1.92	C_29_H_48_O (412)	88	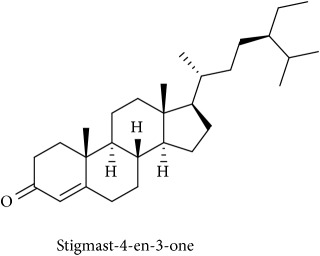

247	19.025	10.51	C_29_H_48_O (412)	95	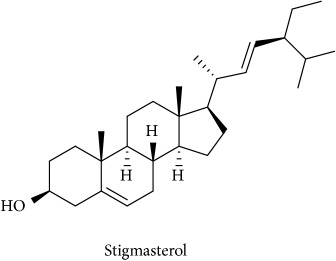

372, 392	15.775	1.51	C_21_H_44_OSi (340)	72	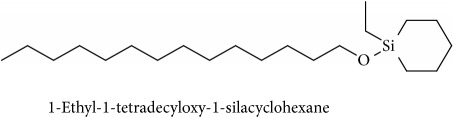

379	19.417	20.20	C_29_H_50_O (414)	94	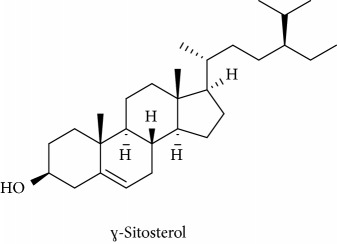

382	19.413	20.20	C_29_H_50_O (414)	78	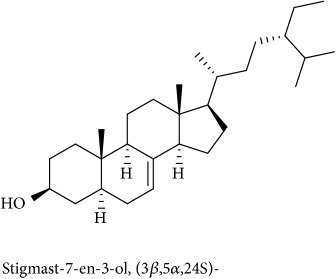

384	15.883	0.75	C_21_H_42_O_4_ (358)	91	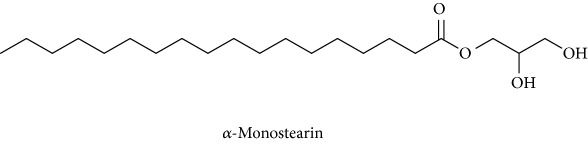

Note: RT = retention time.

**Table 3:** Molecular interaction results of *α*-amylase enzyme protein with the known inhibitor (acarbose) and the bioactive compounds quantified using GC-MS.

Compounds	Binding energy (kcal/mol)	H-bond Interacting Residues	Other Interacting Residues
n-hexadecanoic acid	-2.28	LYS178, TYR174	PRO140, TRP134, SER132, ASP135, LYS172

cholest-8(14)-en-3-ol, 4,4-dimethyl-, (3*β*,5*α*)-	-5.10	-	GLU171, ARG176, ASP173, TYR131, ASP135, SER132, LYS172, TRP134, TYR174

ɣ-sitosterol	-5.28	ASP173	ASP135, GLU171, SER132, TYR131, LYS172, PRO130, TRP134, TYR174

stigmast-7-en-3-ol, (3*β*,5*α*,24S)-	-4.98	-	ASP135, ASP173, SER132, TYR131, LYS172, PRO134, TRP134, TYR174

stigmasterol	-5.35	-	ASP135, ASP173, GLU171, PRO130, SER132, TYR131, LYS172, TRP134, TYR174

acarbose	+1.31	ASP135, LYS172, ARG176	TRP134, SER132, TYR174, ASP173, GLU171

**Table 4:** Molecular interaction results of *α*-glucosidase enzyme protein with the known inhibitor (acarbose) and the bioactive compounds quantified using GC-MS.

Compounds	Binding energy (kcal/mol)	H-bond Interacting Residues	Other Interacting Residues
n-hexadecanoic acid	-4.04	ARG437	ALA444, ASP141, GLN438, GLU432, GLY374, LEU373, LEU433, LYS352, PRO347, THR445, HIS348, PRO351

cholest-8(14)-en-3-ol, 4,4-dimethyl-, (3*β*,5*α*)-	+868.43	-	GLN369, GLU371, GLY370, TYR368, ARG450, LEU45, LEU367, LEU373, LEU446, MET407, PRO408, TRP409, TYR14, TYR41, VAL435, VAL449, PHE21

ɣ-sitosterol	+10.38	PRO347	ALA338, GLN438, GLU432, LEU375, LYS352, PRO351, PRO376, ALA444, ARG437, HIS348, LEU433

stigmast-7-en-3-ol, (3*β*,5*α*,24S)-	+32.55	ARG437	ASN443, ASP441, GLN438, GLU432, GLY374, LEU373, LEU375, THR445, ALA444, LEU433, PRO347, PRO351, PRO376, HIS348

stigmasterol	+74.44	ASP441	ALA349, ASN443, GLN438, GLU432, PRO347, THR445, ALA444, ARG437, HIS348, LEU373, LEU375, LEU433, LYS352, PRO351, PRO376

acarbose	+2547.97	ALA444, GLN438, GLU372, GLY374, HIS348, LEU446, LYS352	ALA434, ASN447, GLN439, GLU371, GLU432, THR448, VAL435, LEU355, PRO351, VAL449

## Data Availability

The data used to support the findings of this study are included within the article.
